# Intelligent and Dynamic Ransomware Spread Detection and Mitigation in Integrated Clinical Environments

**DOI:** 10.3390/s19051114

**Published:** 2019-03-05

**Authors:** Lorenzo Fernández Maimó, Alberto Huertas Celdrán, Ángel L. Perales Gómez, Félix J. García Clemente, James Weimer, Insup Lee

**Affiliations:** 1 Department of Computer Engineering, University of Murcia, 30100 Murcia, Spain; lfmaimo@um.es (L.F.M.); angelluis.perales@um.es (Á.L.P.G.); fgarcia@um.es (F.J.G.C.); 2 Telecommunications Software & Systems Group, Waterford Institute of Technology, X91 K0EK Waterford, Ireland; 3 Department of Computer & Information Science, University of Pennsylvania, Philadelphia, PA 19104-6309, USA; weimerj@seas.upenn.edu (J.W.); lee@cis.upenn.edu (I.L.)

**Keywords:** integrated clinical environments, medical cyber-physical systems, cybersecurity, anomaly detection, ransomware classification, network function virtualization, software-defined networking

## Abstract

Medical Cyber-Physical Systems (MCPS) hold the promise of reducing human errors and optimizing healthcare by delivering new ways to monitor, diagnose and treat patients through integrated clinical environments (ICE). Despite the benefits provided by MCPS, many of the ICE medical devices have not been designed to satisfy cybersecurity requirements and, consequently, are vulnerable to recent attacks. Nowadays, ransomware attacks account for 85% of all malware in healthcare, and more than 70% of attacks confirmed data disclosure. With the goal of improving this situation, the main contribution of this paper is an automatic, intelligent and real-time system to detect, classify, and mitigate ransomware in ICE. The proposed solution is fully integrated with the ICE++ architecture, our previous work, and makes use of Machine Learning (ML) techniques to detect and classify the spreading phase of ransomware attacks affecting ICE. Additionally, Network Function Virtualization (NFV) and Software Defined Networking (SDN)paradigms are considered to mitigate the ransomware spreading by isolating and replacing infected devices. Different experiments returned a precision/recall of 92.32%/99.97% in anomaly detection, an accuracy of 99.99% in ransomware classification, and promising detection and mitigation times. Finally, different labelled ransomware datasets in ICE have been created and made publicly available.

## 1. Introduction

The increasing resilience to antibiotics, an ageing population, the epidemic obesity, or the impact of pollution are factors that increase the difficult for hospitals and care centres to effectively care for patients globally. Hospitals are constantly incorporating technological innovations to face these aspects and improve the quality of the healthcare provided within their borders in the hospital rooms of the future. In this context, new paradigms such as the Internet of Medical Things (IoMT) [1], and Medical Cyber-Physical Systems (MCPS) [2] hold the promise to deliver radical new ways to monitor, diagnose and treat patients through interconnected medical devices with embedded computing systems and networking capabilities.

MCPS refer to safety-critical interconnected medical systems that analyse patients’ vital signs gathered from medical devices, infer the state of the patient’s health, and initiate treatments issuing information to doctors or directly to medical actuators. This disruptive vision has the potential to enable in a cost-efficient way the next-generation of healthcare, which requires systems able to interoperate efficiently, safely, and securely [3]. In this context, the use of interconnected patient-centric medical devices in operating rooms or intensive care units will contribute to reduce human errors and optimize healthcare treatments.

With the goal of making possible the MCPS vision, the ASTM F2761 standard [4] proposes a patient-centric architecture for Integrated Clinical Environments (ICE) that enables the open coordination and interoperability of heterogeneous medical devices and applications. Despite the benefits provided by MCPS and ICE, many of their medical devices and systems incorporating embedded computing and networking capabilities have not been designed to satisfy security requirements and, consequently, are vulnerable to recent attacks [5]. The situation aggravates when medical devices are interconnected to hospital Internet-enabled computer systems. A few years ago, Symantec [6] revealed that more than half of reported outbreaks extended beyond a single medical device, and more than a third of them had experienced a virus or other malware on medical devices, even using protection mechanisms such as firewalls, anti-viruses, or virtual local area networks (VLANs). The Ponemon Institute surveyed in 2016 that 64% of organizations reported a successful attack targeting medical files (9% more than the previous year) and nearly 90% of the attacks to healthcare organizations provoked data breaches [7]. More recently, a Verizon data breach report of 2018 [8] stated that ransomware (malware that encrypts the file system and requests a ransom to decrypt it) accounts for 85% of all malware in healthcare, and more than 70% of attacks confirmed data disclosure. In this context, in January 2018, the Hancock Health Hospital (US) paid attackers $55,000 to unlock systems following a ransomware infection [9]. Previous outbreaks, such as the infamous NotPetya and WannaCry cases in 2017, also affected hospitals worldwide and allegedly forced some of them (16 in the case of the British NHS) to shut down services, send patients to other hospitals and even postpone scheduled surgeries [10].

As it has been demonstrated, ransomware attacks present a critical cybersecurity problem affecting the healthcare discipline. Below, we highlight some of the most important open challenges oriented to the detection and mitigation of ransomware affecting integrated clinical environments [11]:

- Existing protection mechanisms like anti-viruses, intrusion detection systems (IDS), or firewalls are not suitable to detect cyberattacks performed by novel or unseen ransomware. It is due to the fact that their functioning relies on having metadata like, for example, signatures of known cyberattacks.
- Not all the traffic patterns generated by ransomware and malware are distinguishable from the normal traffic patterns generated by medical devices and systems with networking capabilities. In this sense, both a malware encrypting a shared folder and an application compressing the same files have a similar traffic pattern. Similarly, normal changes in the clinical environment can be misinterpreted as attacks if the detection mechanisms do not adapt properly. In addition, malware developers are increasingly using encrypted traffic to avoid payload inspection.
- Achieving an acceptable balance between detection and false alarm rates is a difficult task. A high false alarm rate can be rather frustrating for the administrator and a low detection rate can make the system ineffective.

In this context, Machine Learning (ML) techniques [11] based on the detection of abnormal patterns in the network communications can help to detect unseen ransomware when they spread across the clinical networks or environments. Additionally, when the range of possible classes of anomalies is known and an appropriate labelled training dataset is available, supervised classifiers have obtained satisfactory detection accuracy. On the other hand, the combination of Mobile Edge Computing (MEC) [12] with new technologies as Network Function Virtualization (NFV) and Software Defined Networking (SDN) [13] enables the flexible, cost-efficient, and automatic management of security mechanisms that can allow mitigating the ransomware attacks in ICE.

In order to improve the previous challenges, the main contribution of this article is the design, implementation and validation of an automatic and intelligent system to detect, classify, and mitigate ransomware attacks affecting hospital rooms of the future equipped with ICE. The proposed solution detects and classifies in real time both well-known and unseen ransomware attacks in ICE by analysing the network flows generated during their spreading phase. In addition, once the ransomware has been detected and classified, the proposed mechanism automatically mitigates it by using NVF/SDN techniques to isolate and replace infected devices, avoiding the ransomware spreading across the clinical network. Specifically, we isolate infected medical devices through the SDN paradigm as well as replace their software controllers using NFV techniques. Both the detection and mitigation mechanisms are fully integrated into our previous work, the ICE++ architecture [14]. The ICE++ architecture combines the MEC paradigm with the SDN and NFV techniques to deploy and control in a flexible and efficient way the components making up the hospital room of the future.

Another relevant contribution of this article is a set of experiments that demonstrate the effectiveness of our solution detecting some of the most recent and dangerous malware (namely, WannaCry, Petya, BadRabbit and PowerGhost). In this sense, the selected techniques for both anomaly detection (One-Class Support Vector Machine) and ransomware classification (Naive Bayes) obtained a high precision with known and unseen ransomware samples. Additional experiments demonstrated the viability of the proposed solution in terms of time. In the worst case, our solution detected and mitigated a ransomware attack in less than 30 s, which is an acceptable time because the fastest ransomware required more than 1 min to spread and infect ICE devices. Additionally, as a final contribution, during these experiments, a publicly available labelled dataset was created containing the traffic capture and the resulting netflows from our ICE configuration for both clean and ransomware propagation traffic [15]. In conclusion, the main novelties of our solution are the combination of an anomaly detector and a classifier to improve the detection of ransomware in a clinical environment, the use of multiple flows to compute features and the use of SDN/NFV for mitigation.

The remainder of the paper is structured as follows. Section 2 discusses some related work on security challenges of ICE solutions. Section 3 shows the relevance of ransomware attacks in the hospital rooms of the future equipped with integrated and interconnected medical devices and systems. Section 4 depicts the design details of the proposed detection, classification and mitigation mechanisms. Section 5 shows a summary of the components forming the ICE++ architecture and its integration with our solution. Section 6 shows the experiments performed to demonstrate the viability of our solution. Finally, conclusions and future work are drawn in Section 7.

## 2. Related Work

This section gives an overview of the cybersecurity concerns found in the current state of ICE and MCPS solutions. Historically, medical devices have been developed as stand-alone systems without communication capabilities. However, the MCPS vision is emerging to provide interoperability, safety, and security to clinical environments. In this context, key characteristics in the search for appropriate technology, regulations, and ecosystems to ICE are highlighted in [16]. Nowadays, OpenICE [17] is a commonly adopted implementation of the ICE framework. OpenICE is a distributed patient-centric architecture that implements the components defined by the ICE framework. On one hand, the equipment interfaces can run on computers with limited resources (e.g., BeagleBone Black, Raspberry Pi, etc.), which are physically attached to medical devices to provide network capabilities [18]. On the other hand, the communications between the interfaces and the supervisor are managed by the external DDS middleware [19], which covers partially the cybersecurity of OpenICE.

In the literature, there are different solutions oriented to analyse and mitigate the security and privacy issues of healthcare and MCPS [20]. In this context, one of the most recent works is presented in [21], where the authors review the major security techniques available in the state of the art and study their applicability and utility for the design of MCPS. After that, the authors define an abstract architecture for MCPS to demonstrate various threats. In [22], a prototype is developed to protect the communications of solutions based on the ICE framework through the security mechanisms provided by the Object Management Group (OMG) Data Distribution Service (DDS) standard [19]. Specifically, the DDS middleware adds support for authentication, authorization, access control, confidentiality, integrity and non-repudiation of the exchanged information. After several experiments, the authors state that transport-level security (TLS) solutions may not provide sufficient resilience against insider attacks, while DDS potentially addresses or mitigates disturb, eavesdrop, and denial of service (DoS) attacks. Despite the important outputs of this proposal, it is not clear how DDS is able to mitigate DoS attacks. Another work, oriented to protect the security and privacy of solutions based on ICE, is proposed in [23]. The authors of this work propose a cloud-based secure logger that receives the information sensed by ICE interfaces attached to medical devices. The proposed logger relies on standard encryption mechanisms to maintain a secure communication channel even on an untrusted network and operating system. This solution is effective against replay, injection, and eavesdropping attacks. However, any behaviour that does not lead to message alteration is not detected. In [24], it is designed and implemented an authentication framework for ICE-compliant interoperable medical systems. The proposed framework is composed of three layers, allowing it to fit in the variety of authentication requirements coming from different ICE entities and networking middlewares. The performed experiments demonstrate that the proposed authentication framework protects OpenICE against device replacement and impersonation attacks.

In addition to the previous security mechanisms proposed in ICE, in the literature, we can find solutions in heterogeneous scenarios that leverage SDN to detect/mitigate ransomware attacks, some of them using different ML techniques for detection purposes. However, to the best of our knowledge, none of them has integrated the combination SDN/NFV with the ICE standard to be able to replace infected elements such as ICE Equipment Interfaces or ICE Supervisor in a few seconds. Moreover, the detection of the ransomware spreading in ICE with encrypted traffic has not been addressed yet.

Among the ransomware mitigation solutions based on SDN, Ref. [25] uses SDN redirection capabilities together with a blacklist of proxy servers to check if the infected device is trying to connect to one of them to obtain the public encryption key. The mitigation consists of establishing a flow filter to impede this communication and, thus, the encryption of the files. The main drawback of this proposal is that it needs to keep a blacklist of proxy servers updated. These servers must be identified by means of behavioural analysis of known malware, thus making it impossible to detect new campaigns. When compared with our solution, our goal is not to prevent the encryption of the files. We attempt to detect the ransomware spreading by using the characteristic traffic patterns generated during that stage. Additionally, our mitigation procedure restores the ICE system to a clean state.

Another relevant and recent paper related to SDN and ransomware is [26]. In this work, the authors use deep packet inspection to identify HTTP POST messages, defining as feature vector the lengths of each three consecutive HTTP POST messages. Then, a classifier is trained by computing both the centroid of the feature vectors belonging to each ransomware class, and a maximal distance to be considered from that class. They obtain an false positive ratio (FPR) of about 4% using this method. The main difference with our proposal is that the latter one does not inspect the payload, thus being suitable even with encrypted traffic. Additionally, our combination of anomaly detector and classifier reaches an FPR of less than 1%. Unfortunately, these results are not comparable due to the context of each solution.

In [27], the authors recognise that malware developers are increasingly using encrypted traffic to avoid payload inspection. Therefore, they propose using SDN to obtain flows and compute a feature vector made up of a combination of interarrival times, packet ratios and burst lengths. They train a random forest to detect the traffic exchanged between the infected device and the Command & Control (C&C) server, obtaining around 10% of FPR. Our solution aggregates the flows belonging to a given time window, thus obtaining more expressive features. Although our FPR is virtually zero, the results are not comparable due to the different contexts.

Regarding other types of malware, the authors of [28] proposed a system able to detect it using machine learning classifiers. They focused on analysing the network traffic and selected four categories of features: basic information, content based, time based and connection based. To perform the system evaluation, the authors used two different datasets, concluding that Bayes network and random forest classifiers produced more accurate outputs than other ML solutions like multilayer perceptron. Similarly, in [29], deep learning techniques are proposed to detect botnet attacks by analysing network flow patterns from real botnet traffic captures. This is carried out in the context of a MEC oriented system based on NFV/SDN that provides a dynamic management of the resources involved in the detection process. Another solution was presented in [30], where the authors performed an ML analysis of ransomware affecting the MS Windows operating system. They considered the network traffic to achieve an acceptable detection rate. A dataset created from conversation-based network traffic features was used to achieve a true positive detection rate of 97% using the Decision Tree (J48) classifier. The proposal presented in [31] also was focused on ransomware detection. Unlike the previous solutions, this approach extracted relevant features from the API call history produced by ransomware attacks. These features were used along with a Support Vector Machine (SVM) to detect unknown ransomware. The authors demonstrated a correct detection ratio by considering 276 ransomware samples in a Sandbox. The authors of [32] developed a network intrusion detection model based on big data and netflows. In addition, six feature selection algorithms were combined to achieve a better accuracy in terms of classification. On the other hand, in [33], a methodology for trusted detection of ransomware in a private cloud is presented. The authors considered volatile memory dumps of virtual machines to create features. The results showed that the proposed system was able to detect anomalous states of a virtual machine, as well as the presence of both known and unknown ransomware. Table 1 compares our solution with the most relevant ones of the state of the art by considering different criteria.

The previous solutions improve in a proactive and reactive way the cybersecurity challenges of clinical and heterogeneous environments. However, they do not consider the real-time and autonomic detection and mitigation of ransomware during its spreading phase. Additionally, these solutions are not integrated into an adaptive and flexible management architecture, which is able to manage the security mechanisms according to the contextual information (for example, when the ICE system is under a given attack). To the best of our knowledge, this paper proposes the first solution combining ML techniques to detect and classify ransomware as well as the use of NFV/SDN techniques combined with MCPS to provide a flexible management of the ICE resources in real time and on-demand.

## 3. Ransomware in the Hospital Rooms of the Future

This section presents a clinical scenario that shows real issues caused by a ransomware attack affecting hospital rooms of the future. For the sake of clarity, we focus the explanation on the behaviour of the WannaCry ransomware family. However, it is important to note that the solution proposed in this paper is generic enough to detect and mitigate other families of ransomware, as it has been demonstrated in Section 6.

### 3.1. The Hospital Room of the Future

**Figure 1:**
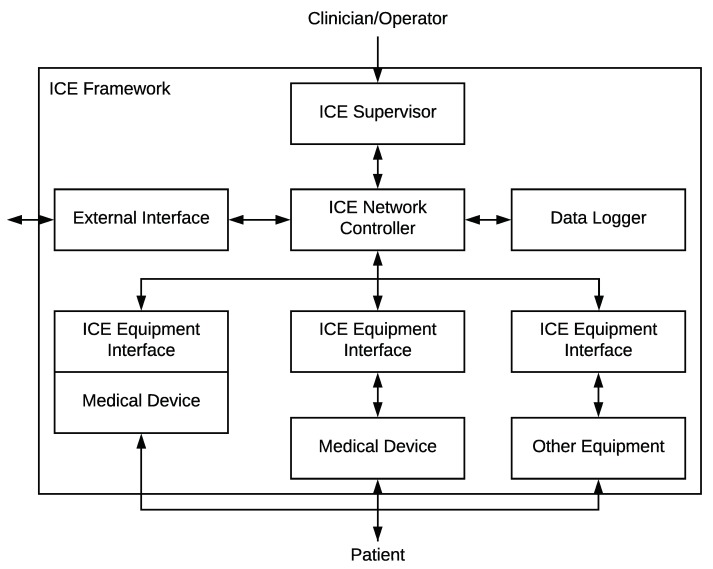
Elements composing the integrated clinical environment (ICE) framework.

With the goal of facilitating the interoperability of the elements comprising the hospital rooms of the future and making possible the MCPS vision, the ASTM F2761 standard [4] proposes a patient-centric architecture for ICE that enables the open coordination of heterogeneous medical devices and applications. Among the proposed components of the ICE framework, the most relevant are the *ICE Equipment Interfaces*, which are attached to medical devices to enable their networking capabilities; the *ICE Supervisor*, focused on hosting medical applications that receive and control the patients’ vital signs; the *ICE Network Controller*, in charge of enabling the communications between the supervisor and the ICE equipment interfaces, as well as handling and maintaining the discovery of medical devices and their information; the *Data Logger*, focused on troubleshooting and forensic analysis; and *External Interface*, which enables the communication with external hospital resources such as Electronic Health Records (EHR). Figure 1 shows the elements composing the ICE framework and their communications.

### 3.2. WannaCry Ransomware Infecting and Spreading across the Hospital Room of the Future

Over the last few years, ransomware has gained relevance as a devastating method of cybercrime in healthcare environments. Locky [26], SamSam [34] and, more recently, WannaCry [35] and Petya [36] are well-known families of ransomware that have affected hospitals worldwide and allegedly forced some of them to shutdown services, send patients to other hospitals and even postpone scheduled surgeries.

To contextualize, ransomware is a type of malware that encrypts the files of infected devices (affecting the availability of data and/or device) and requests a ransom to decrypt the files and avoid information losses. Recent ransomware attempts to exploit system vulnerabilities to spread out rapidly through the networks and affect as many devices or machines as possible. This propagation process is much more harmful when the number of systems is significantly larger. Nowadays, there are several families of ransomware with important differences in terms of encryption and spreading behaviours. One of the most recent and malicious families is WannaCry. This ransomware spreads automatically across the network by exploiting a vulnerability in the MS Windows based Server Message Block (SMBv1). Despite this vulnerability having already been fixed by Microsoft, it is critical to have mechanisms able to detect unseen or known ransomware exploiting well-known or undetected vulnerabilities. Otherwise, hospital rooms of the future equipped with a large number of interconnected medical devices and computational systems will suffer ransomware attacks, affecting the patients’ safety and privacy.

As an example and to illustrate the ransomware impact in the hospital room of the future, we consider that the ICE Supervisor becomes infected by WannaCry. As it has been demonstrated at the beginning of this section, this situation is not unreal and it can happen due to plugging external devices with malicious or infected files, social engineering, weaponized files, or phishing. Once the ICE Supervisor is infected, WannaCry attempts to connect to a predefined domain. If the site was registered, the ransomware is not executed, acting as a killswitch. In contrast, if the connection fails, the ransomware starts with the following two phases:

- Spreading phase: The WannaCry ransomware attempts to spread itself across the hospital network to infect vulnerable medical devices and computers. For that goal, WannaCry uses EternalBlue, an exploit developed by the National Security Agency (NSA) that attacks a vulnerability of the MS Windows based Server Message Block (SMBv1) protocol. After a successful exploitation, the DoublePulsar payload is sent to run remote code and infect the medical devices and computers connected to the ICE network.
- Encryption phase: It can be performed before, in parallel, or after the spreading phase (depending on the ransomware version and family). During this phase, the medical database as well as other data files are encrypted. Once the process is completed, WannaCry asks for a ransom to decrypt the medical database. Ransomware belonging to the WannaCry family generates and saves a new RSA key pair, which is used to encrypt the medical database and the target data files. Once the previous files are encrypted, WannaCry deletes the original ones, and communicates with an Onion server using a Tor server to transfer the encryption keys. When the ransom is paid, WannaCry obtains the decrypted RSA private key from the Onion server and decrypts the ransomed files.

As it has been explained in Section 2, current ICE security solutions do not have capabilities to detect and mitigate the spreading phase of WannaCry or other ransomware, which supposes a critical cybersecurity issue. During the process of detecting ransomware, it is mandatory to have an efficient mechanism to monitor and analyse in real time the network traffic and identify anomaly communications between the medical devices connected to the ICE network. Additionally, it is also critical to have an autonomic mechanism able to react and mitigate ransomware attacks once the infection has started. This mechanism should be able to perform different tasks like stopping the spreading phase by dropping infected network packets, and replacing the infected software. To reach both detection and mitigation capabilities, a solution like the proposed in this paper is required.

## 4. Design of Our System to Detect, Classify and Mitigate Ransomware in ICE

This section presents the design details of our intelligent and automatic solution to detect, classify and mitigate ransomware attacks in hospital rooms of the future. Having in mind the clinical scenario defined in Section 3, the aim of our approach is twofold. On the one hand, we want to detect anomalies or novelties in the network traffic patterns produced by ransomware spreading across hospital rooms of the future equipped with ICE. To this purpose, a semi-supervised anomaly detection method is especially well suited to detect unknown ransomware, when network traffic generated during its propagation phase is sufficiently different from normal traffic patterns. On the other hand, we want to provide some feedback to the administrator about the nature of the propagating ransomware. Therefore, a probabilistic supervised ransomware classifier can give some identification support to our anomaly detection system. The main reason for using ML techniques instead of heuristics or indicators of compromise is the ability of the former to both extract complex patterns from the data and generalise to unseen samples. This ability is essential to find hidden patterns in statistical features computed from flows.

**Figure 2:**
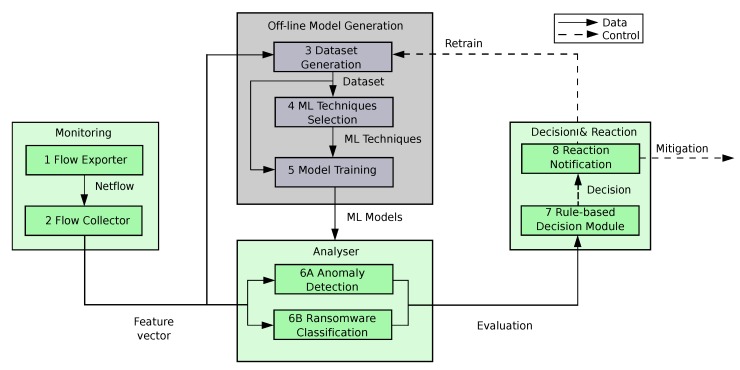
Design of the proposed solution to detect, classify and mitigate ransomware in ICE.

The following subsections, together with Figure 2, show the modules, components and life-cycle of our solution. In addition, below we provide an overview of the main modules making up the proposed system.

1. The Monitoring module is in charge of acquiring in real time the network traffic generated by medical devices and systems belonging to ICE, calculating network flows, grouping them in a time sliding window and computing a feature vector from each group or batch of flows.
2. The Offline Model Generation module, in contrast to the rest of modules, requires human supervision. It is only executed during the system bootstrapping, or when it is required to update the existing ML models due to the detection of a new ransomware. This module receives feature vectors for a given period of time (usually a few hours), generates a dataset suitable for the training process, selects the proper ML algorithms to detect and classify ransomware in ICE, and trains those algorithms.
3. The Analyser module receives the trained ML models from the previous module and uses them to evaluate in real time the current feature vectors. The anomaly detection method will identify traffic pattern novelties (potentially coming from ransomware attacks), whereas the classifier will label the traffic coming from known ransomware.
4. The Decision & Reaction module combines the evaluations of the two ML models to estimate the risk of having an active ransomware attack. For that, it uses rules, predefined by the system administrator, to decide in real-time proper mitigation actions like, for example, the isolation and replacement of infected medical devices, or the retraining of ML models.

### 4.1. Monitoring: Generating Network Flow Features in ICE

This is the first module of our system and focuses on monitoring in real time and continuously network packets exchanged between the medical devices and databases of ICE. After that, network packets are processed and translated to network flows. The main reason for using network flows is because some pieces of clinical data travel encrypted, even certain ransomware cyphers its spreading traffic, which makes it useless to inspect the network packets. In this point, it is important to consider that we define a network flow as all the traffic exchanged in both directions, during a given connection sharing the same values of source IP, destination IP, source port, destination port and protocol.

#### 4.1.1. Flow Exporter

The Flow Exporter (step 1 in Figure 2) is a component of the monitoring module that captures the network traffic and generates a record for each flow in starting time order, containing statistical data about its associated traffic. We use the Network flow format (Netflow), which is a well-known flow format created by Cisco and has become a standard in the industry. Our specific flow record format differs slightly from the usual bidirectional Netflow record by including the ARP protocol (only the meaningful fields have been used in this case). Table 2 lists the fields considered by our proposal.

Due to the definition of network flow, there can be a large number of packets belonging to the same flow, e.g., when doctors read a big file stored in the medical database, or when UDP packets with patients’ vital signs are exchanged between medical devices (ICE Equipment Interfaces) and the medical application (ICE Supervisor). In these cases, our flow collector is configured to generate a new network flow periodically (triggered by a timeout that prevents a large flow from appearing only once), which would affect the performance and detection time of our solution (see Section 6). In our system, the timeout is determined by the Flow Collector configuration in order to guarantee that each large flow is included in the Flow Collector computations during its whole life.

#### 4.1.2. Flow Collector

**Figure 3:**
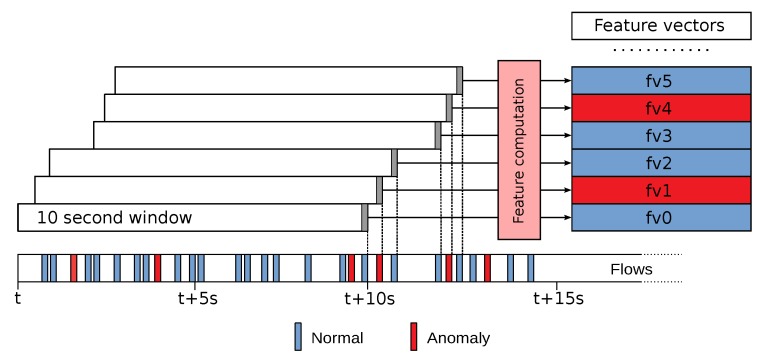
Feature computation. Every time a new flow arrives, the flows received during the last 10 s are used to compute the aggregated features. The feature vector is created by assembling these aggregated features plus some additional ones obtained from this last flow. The feature is labelled as anomalous only if the last flow is anomalous.

The Flow Collector (step 2 in Figure 2) is the other component of the monitoring module. It retrieves (and optionally stores) the Netflows calculated by the flow exporter and computes feature vectors from them. Since a single Netflow record is too simple and only provides few features, we propose the use of sliding windows of Netflows. Every time a new Netflow is acquired, an aggregation process is triggered to create a vector of aggregated features using the set of flow records obtained for the last 10 s (justified in Section 6), plus some additional features extracted from the last flow. Figure 3 illustrates the feature generation procedure for our time window of 10 s and the list of computed features is described in Table 3. These features are intended to measure a wide variety of traffic pattern parameters. These parameters are considered relevant to model both the normal traffic of ICE and the particular traffic perturbations that ransomware spreading produces. In this manner, our feature vector contains context features (computed from the window of flows) and local features (computed from the last flow). Among the context features, some of them are computed from the flows sharing the same protocol (TCP/UDP/ARP) in a given sliding window and others are computed from the sliding window flows that share the same protocol and direction. Direction can be internal/external (both source and destination IPs belong to the ICE or are external to the ICE) or incoming/outgoing (external IP started a flow towards an internal IP and vice versa).

The functionalities of both the flow exporter and collector have been deployed in the Monitor component of the ICE++ architecture (illustrated in Section 5).

### 4.2. Offline Model Generation: Selecting and Training ML Models

**Figure 4:**
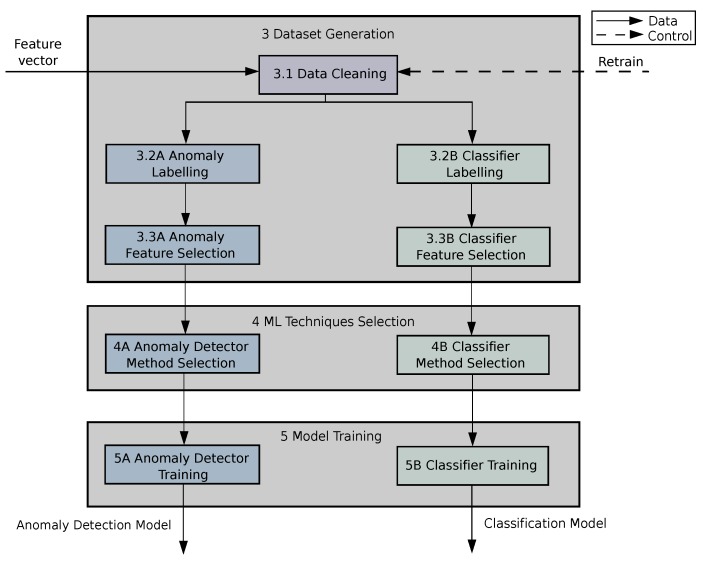
Design of the Offline Model Generation module to detect and classify ransomware in ICE.

This module encompasses the whole process of model generation as a fundamental initial stage to provide the Analyser with a pair of well-suited ML models to detect and classify known and unseen ransomware attacks in our hospital room of the future (step 3 of Figure 2). In contrast with the other modules, it is not performed in real time and needs human supervision to process the following steps: obtaining a clean labelled dataset that models the behaviour of the ICE with a subset of most discriminative features, selecting a pair of proper ML techniques (anomaly detection and classifier) and finally training them with the previous dataset. The following subsections together with Figure 4 show in detail the processes making up the module.

#### 4.2.1. Dataset Generation

The main goal of the Dataset Generation component (step 3 in Figure 4) is to create a suitable group of feature vector datasets: one dataset generated with normal traffic acquired from a clean ICE to train the anomaly detector, and as many datasets with infected ICE traffic as different ransomware samples we want to classify, in order to train the supervised classifier.

The initial set of features (detailed in Table 3) provided by the flow collector is created with the aim of providing an extensive set of predictors which could be subsequently refined by means of feature selection techniques. Since some of the feature vectors can include invalid, constant, or highly correlated features, the datasets have to be cleansed to repair/discard wrong feature values and remove all the features with zero variance (constant). As an illustration, NaN values or empty ports are converted into zero, and if a feature has a constant value, the feature is discarded. Additionally, certain features need to be transformed to improve their value distribution, e.g., applying a log function to equalize domain ranges and make the histogram more Gaussian. This Data Cleaning process is carried out in step 3.1 of Figure 4.

The next step (3.2) is to label the datasets according to its purpose, and to divide them into training, validation and test. This is clearly necessary in the case of our supervised classification method. On the other hand, for the anomaly detection method, there is no need of an explicit labelling for the training. However, in this case, it is required an explicit labelling to evaluate the performance of both the detector and the classifier. More specifically, the anomaly detector needs a binary label (normal/anomaly), whereas the classifier needs as many labels as classes. Figure 3 illustrates that the label of each feature vector is determined by the last flow used to compute it. Therefore, the feature vector associated with a given flow is made up of both data from this flow and statistical data obtained from a set of previous flows, thus providing additional traffic context. As a consequence, the label of a feature vector is the label of its last flow, that is, the traffic class to which the flow belongs.

Subsequently, a first feature selection is done by computing the Pearson’s correlation coefficient for each pair of components in the feature vector. This correlation matrix of the dataset allows us to identify sets of highly-correlated features and keep just one feature of each set. This procedure is done in both 3.3A and 3.3B steps. An additional feature selection stage could reduce significantly the number of features of the datasets. However, this phase is not carried out on the datasets used with the anomaly detection methods. The main reason is that a more aggressive feature selection procedure based on the already-known ransomware classes could discard features useful for detecting future anomalies. This additional feature selection is only carried out on the dataset for the supervised ransomware classifier (3.3A). To this end, we use a Random Forest (RF) [37] supervised classifier. This algorithm has the property of computing an information score for each feature during the training, which can be used to obtain a subset of the most informative features. Every feature with an importance score below a given threshold can be discarded.

#### 4.2.2. ML Technique Selection

This component is in charge of determining the most suitable detection and classification techniques for ICE in terms of performance (steps 4.A and 4.B). For that goal, below we show the variety of initial methods considered for both classification and anomaly detection.

- Anomaly detection techniques
  – One-class Support Vector Machine (OC-SVM), [38]
  – Local Outlier Factor (LOF), [39]
  – Isolation Forest (IF), [40]
- Probabilistic classification techniques [41]
  – Neural Network (NN),
  – Naive Bayes (NB),
  – Random Forest (RF).

Regarding the anomaly detection (step 4.A), our system uses the OC-SVM technique. OC-SVM belongs to the class of boundary-based classification algorithms. The training process of this method consists of finding a function which defines a boundary surrounding the normal traffic patterns of our ICE. This function returns a positive value for points belonging to the inner area defined by the boundary, and a negative value for outsiders. The anomalous traffic pattern generated by the ransomware propagation ought to fall in the negative side.

On the other hand, with regard to the probabilistic classification techniques (step 4.B), the goal is to determine to what extent they can deal with ransomware sharing key spreading aspects with the samples used in training. In this sense, probabilistic classification methods, whose output is an estimation of the likelihood of the vector belonging to each class that can help to reach this goal. Even if an input vector does not belong to any known class, the probability distribution provided by the algorithm can give some useful information to the system administrator about the behaviour of the potential malware. In this sense, the proposed system uses Naive Bayes because it provided the best probability estimation of the belonging of the new samples to the class with the most similar traffic pattern. Section 6 presents some experiments and explanations that justify the selection of both anomaly detection and classifier techniques.

#### 4.2.3. Model Training

Once the best ML techniques have been selected, this component is in charge of re-training the ML techniques (anomaly detection and classification) with the whole dataset (not only the training subset) in order to obtain a model that takes advantage of all the samples available. These processes are performed by steps 5.A and 5.B of Figure 4, the resulting models being sent to the *Analyser* module.

In the anomaly detection model case (step 5.A), an automatic retraining process can be triggered if the configuration of the ICE changes significantly (e.g., installation of new devices or activation of new services/protocols). On the other hand, the supervised classifier needs a human-driven dataset generation process to be updated.

### 4.3. Analyser: Detecting Anomalies and Classifying Ransomware Attacks in ICE

The Analyser is the module that evaluates in real time each incoming feature vector from the medical devices and systems of ICE by means of previously trained models (anomaly detector and classifier). The output of the anomaly detection (step 6A of Figure 2) is twofold: a score of the anomaly degree of the sample, and a binary prediction as anomalous or normal according to a standard threshold of 0.5. On the other hand, the classifier (step 6B), has also two outputs: an estimation of the probability of belonging to the normal traffic and an array with an estimation of the probability of belonging to each ransomware class. The outputs of both methods are sent to the *Decision & Reaction module* which will interpret the results and will react in consequence to mitigate the potential ransomware attack.

### 4.4. Decision and Reaction: Mitigating Ransomware Attacks

This module automatically makes decisions and notifies different components to react and mitigate the spreading phase of ransomware infecting our ICE. It is made up of the following two modules: the Rule-based Decision and the Reaction and Notification.

#### 4.4.1. Rule-Based Decision

The Rule-based Decision component (step 7) allows the system administrator to predefine rules that control in real time and on-demand the mitigation reactions. These rules consist of two lists of predicates: the antecedent and the consequent. If all the predicates of the antecedent part take the Boolean value true, all predicates in the consequent part are evaluated. The antecedent considers conditions related to the Analyser outputs and the consequent indicates the action taken over the datasets, NFV or SDN paradigms to mitigate the ransomware.

In the context of our hospital rooms of the future equipped with ICE, we define three types of mitigation rules: Control plane, Data plane and Hybrids.

- Control plane rules are used to manage the different components and modules making up the control plane of the ICE++ architecture. Among the potential actions taken by these policies, we highlight the retraining of ML models when unseen ransomware strains are detected, the set-up of new time windows, or the management of the architecture hardware resources. As an example, the next rule retrains the Anomaly Detector ML model when a new medical device is integrated into ICE. In this particular situation, the rule indicates that the anomaly detector needs to be retrained if it has detected an anomaly and the classifier considers that it is more likely that the vector belongs to normal traffic:
  $$Anomaly(v)\;\&\;\left[P_{N}\left(v\right)-max\left(P_{R}\left(v\right)\right)>epsilon\right]\;\to \;\mathrm{Anomaly}\;\mathrm{detector}\;\mathrm{retrain},$$
  where *v* is the feature vector evaluated by the two models; Anomaly(v) is the boolean output of the anomaly detection module for that input; epsilon is a threshold defined by the administrator; the ransomware classifier output is represented by $P_{R}\left(v\right)$ and $P_{N}\left(v\right)$, $P_{N}\left(v\right)$ being the estimated likelihood of being a normal behaviour, and $P_{R}\left(v\right)$ the array containing the likelihood estimation for each trained ransomware class.
- Data plane rules are oriented to control the medical devices, systems, EHR, and medical applications of ICE. Potential reactions could be the isolation of infected medical devices or systems, the replacement of medical controllers, or the communication management. As an example, the next rule indicates that a medical device must be isolated and replaced if an anomaly has been detected and the classifier certainly believes that the vector belongs to one of the known ransomware. In other words, the ICE is experiencing a propagation stage of a ransomware sufficiently similar to one of the known samples:
  $$\begin{array}{c} \;Anomaly(v)\;\&\;\left[max\left(P_{R}\left(v\right)\right)-P_{N}\left(v\right)>epsilon\right]\;\&\\ {}[max\left(P_{R}\left(v\right)\right)-submax\left(P_{R}\left(v\right)\right)]>lambda\;\to \;\mathrm{MD}\;\mathrm{Isolation}\;\&\;\mathrm{Replacement}, \end{array}$$
  where submax() returns the element immediately below the maximum, lambda is a threshold defined by the administrator and represents the certainty about the predicted ransomware, and MD is the infected medical device.
- Hybrid rules are a combination of the two previous families. The consequence of these rules implies changes in both control (ICE++ Architecture) and data hospital rooms of the future devices and systems. The next rule shows an example where it is required to isolate and replace a medical device as well as retrain the classifier model. In this case, the Anomaly Detector says that there is an anomaly and the Classifier is not able to establish a classification with enough guarantees. This situation is provoked by an attack conducted by an unseen ransomware, which behaviour is not similar to any of the well-known ones:
  $$\begin{array}{c} \;Anomaly(v)\;\&\;\left[max\left(P_{R}\left(v\right)\right)-P_{N}\left(v\right)\leq epsilon\right]\;\&\\ \;[max\left(P_{R}\left(v\right)\right)-submax\left(P_{R}\left(v\right)\right)]\leq lambda\;\to \\ \mathrm{MD}\;\mathrm{Isolation}\;\&\;\mathrm{Replacement}\;\&\;\mathrm{Classifier}\;\mathrm{model}\;\mathrm{retrain}. \end{array}$$

#### 4.4.2. Reaction and Notification

**Figure 5:**
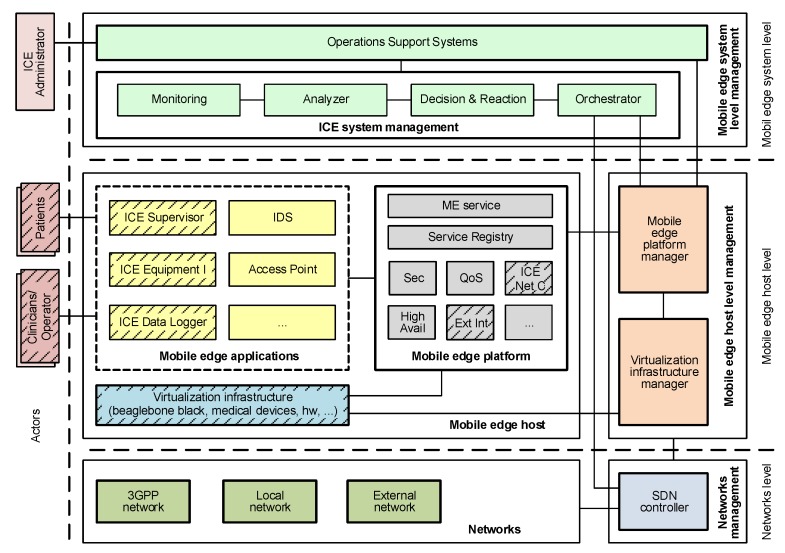
ICE++ architecture oriented to the Mobile Edge Computing (MEC) paradigm.

The Reaction and Notification component (step 8) is in charge of interacting with two different modules. On the one hand, it interacts with the Offline Model Generation module when it is required to retrain a given ML module. On the other hand, it interacts with the Orchestrator module (see Figure 5) to schedule the enforcement of the decided mitigation actions when a ransomware has been detected. Finally, this module also creates and maintains a log where the different reaction decisions are stored for analysis purposes. The components indicated by the steps 7 and 8 have been deployed in the Decision and Reaction module of the ICE++ Architecture (see Section 5).

## 5. ICE++ Architecture

This section describes the ICE++ architecture, and how it integrates the modules making up the proposed solution to detect, classify, and mitigate ransomware in ICE (explained in Section 4). In other words, in this section, we will see how the anomaly detection and classification techniques depicted in Figure 2 are used by our architecture to detect ransomware attacks affecting the elements of Figure 1.

ICE++ combines the MEC, SDN, and ETSI NFV [42] proposals to enable the flexible, efficient, and automatic management of the elements composing the ICE standard. Figure 5 depicts the layers and actors of ICE++ as well as how the ICE elements are provided. These elements are depicted in boxes with striped background to show clearly how the ICE++ architecture interacts with the existing ICE framework. The internal communications between the ICE elements are not depicted for better understanding (they are shown in Figure 1).

### 5.1. Mobile Edge System Level

*Mobile edge system level management* is in the upper level of our architecture and it is focused on defining and managing the behaviour of the ICE components. For that, this level is made up of the *Operation Support System* (OSS) and the *ICE System Management*. The OSS deals with the logic of the ICE system. This element provides the system administrator with an interface to define the rules controlling the architecture behaviour. On the other hand, the rules are provided to the *ICE system management* in order to identify concrete actions and orchestrate their enforcement.

The ICE system management is composed of the next elements: *Monitoring*, *Analyser*, *Decision and Reaction*, and *Orchestrator*. They are in charge of detecting and mitigating ransomware attacks in ICE. Section 4 depicts in detail our design for the first three components, and the orchestrator is explained in this section. In a nutshell, the monitoring component gathers in real time the network packets, generates network flows and finally feature vectors. These vectors are sent to the analyser, which uses ML techniques (anomaly detection and classification) to decide if there is some ransomware affecting the ICE. Once detected the ransomware, the decision and reaction component decides the proper countermeasure to mitigate the attack according to predefined rules. Among the set of potential countermeasures, we highlight the flexible and efficient deployment, configuration, relocation, and dismantlement of:

- ME applications: ICE components (interfaces, supervisors, or applications) to replace infected systems of the hospital room of the future.
- ME services: For example, an ME service could be in charge of assuring the level of security of the ICE elements. In particular, it could establish specific authentication mechanisms to ICE applications or determine a particular security protocol (e.g., TLS) between the ICE elements.
- Network infrastructure: For example, virtual SDN-based networks to isolate infected ICE components. Another alternative could be the automatic management of the network communication to block ransomware attacks.

Finally, the Orchestrator is responsible for scheduling and triggering the previous countermeasures as well as maintaining an overall view of ICE status (ME hosts, available resources, and network topology).

### 5.2. Mobile Edge Host Level

This level focuses on running the mitigation countermeasures (ME applications, services, and virtualized infrastructure) in ICE. This level is composed of two elements: the *Mobile edge host* and the *Mobile edge host level management*.

On the one hand, the ME host provides ICE with compute, storage, and services for running ME applications. To reach it, this entity contains *Mobile edge applications*, a *Mobile edge platform*, and a *Virtualization infrastructure*. ME applications could be instantiated as the ICE element defined by the ICE framework as well as some applications oriented to improve the security of the clinical scenario. Applications run as virtual machines (VM) and containers on top of the virtualization infrastructure allocated at the edge of the network. This fact provides our solution with the flexibility, efficiency, and low latency required by ICE. The virtualization infrastructure can use the hardware resources of computers, BeagleBones Black, or even medical devices, depending on the scenario configuration. ME applications interact with the ME platform to consume and provide services. Specifically, the ME platform is a set of essential services required to run ME applications on a particular virtualization infrastructure. These services can be specific for given applications or even shared among some of them. Examples of services could be secure communication protocols (like Transport Layer Security) or traffic rules control.

On the other hand, the ME host level management is composed of two elements: the *Mobile edge platform manager* and the *Virtualization infrastructure manager*. The ME platform manager is responsible for managing the life cycle of applications, including informing the ME orchestrator of relevant application related events. The Virtualization Infrastructure Manager is responsible for allocating, managing and releasing virtualized (compute, storage and networking) resources of the virtualization infrastructure, as well as collecting and reporting performance and fault information about the virtualized resources.

### 5.3. Networks Level

Finally, the *Networks* level is the lowest one and it contains two elements: the *Networks* and the *Networks management*. Networks contain the physical infrastructure required to provide connectivity between the different ME applications. The Networks management contains the SDN Controller, which is able to monitor and manage in real time and on-demand the communications of ME applications.

### 5.4. Actors Level

The actors composing the proposed architecture are: *ICE Administrator*, *Clinicians & Operators*, and *Patients*. The ICE Administrator is responsible for defining the logic of the system in a high level. The rules defined in Section 4 are examples that indicate when, how and where it is required to react and mitigate a given ransomware attack. The clinicians and operators interact with the ME applications (ICE applications, access points, etc.), for example, to obtain the patients’ vital signs or the state of the active treatments. Finally, patients, whose vital signs are monitored by the medical devices, can also interact with the ME applications to obtain medical and personal information like, for example, Electronic Health Records (EHR).

### 5.5. Enforcing a Ransomware Mitigation in ICE

Once the main components of the architecture has been explained, this section shows how the *Orchestrator* enforces the mitigation actions taken by the second rule defined in Section 4.

**Figure 6:**
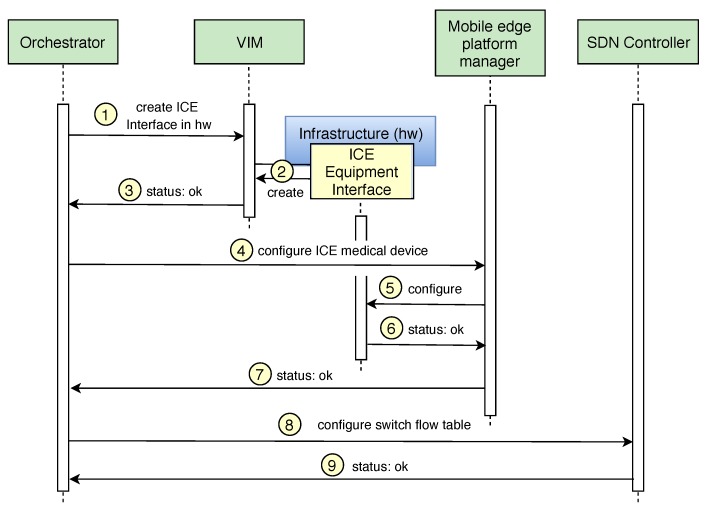
Sequence diagram to mitigate a ransomware attack to a medical device.

Regarding the enforcement of the rule in charge of replacing and isolating the infected medical device, the Orchestrator receives the notification and interacts with the virtualization infrastructure manager (VIM) to create the new ICE Equipment Interface in the available hardware located in our hospital room of the future (step 1 of Figure 6). Once the VIM receives the request, it checks the available virtual and physical resources and creates a new Virtual Machine or Docker with the ICE Equipment interface in the existing infrastructure (step 2). The action is communicated to the Orchestrator component (step 3), which updates the catalogues with the information of the new ICE component. Once the new ICE Equipment Interface has been deployed, the Orchestrator interacts with the mobile edge platform manager (step 4) to provide the configuration of the virtual medical device instantiated in the ICE Equipment Interface. Several parameters are established in this configuration such as the IP address of the ICE Supervisor, the frequency of communication, etc. After that, the mobile edge platform manager configures the ICE Equipment Interface. Finally, the status is notified to the Orchestrator (steps 6 and 7).

Once the new medical device starts monitoring the patient’s vital signs, the Orchestrator enforces the isolation of the infected device by filtering the network packets belonging to the spreading phase of the ransomware attack. Here, it is important to mention that, depending on how critical the medical device function is, the next process can be made before of after its replacement (steps from 1 to 7). In this point, the Orchestrator interacts with the SDN Controller to add a new rule in the flow tables of the network switch and drop the packages belonging to the spreading phase of the ransomware (step 8). Finally, the SDN controller confirms the Orchestrator that the flow tables have been modified with the new rule (step 9). The time required to perform the deployment of a new ICE Equipment interface in different hardware resources has been calculated in Section 6 through different experiments. On the other hand, the time to isolate the infected device (steps 8 and 9) is negligible (ms) in our context.

## 6. Experiments

This section presents a pool of experiments focused on demonstrating the viability of our intelligent and automatic solution in charge of detecting, classifying and mitigating ransomware attacks in hospital rooms of the future.

There are two main aspects that should be considered to measure the viability of our proposal: how good our system is at detecting ransomware threats and how effective our system is at mitigating a ransomware propagation. The detection aspect is tightly related to the discriminative power of the trained ML algorithms. On the other hand, the mitigation aspect is related to both the time needed to detect the propagation and the ability to mitigate the ransomware before it manages to infect other medical devices and systems. To measure both aspects, firstly we have designed and deployed a realistic scenario where a hospital room of the future equipped with ICE is infected with different ransomware. After that, we performed some experiments to measure the detection and classification precision as well as the time required to mitigate the ransomware. The experiments were carried out on OpenICE, the open source implementation of the ASTM F2761 (ICE) [4], proposed by the MD PnP Medical Device Interoperability Program of the Massachusetts General Hospital Department of Anesthesia, Critical Care, and Pain Medicine. Their work on safe interoperability spans the entire healthcare provision, and the ICE standard is strongly influencing the trajectory of medical device interoperability in the U.S. All of this support guarantees its applicability.

### 6.1. Deployment and Configuration of Our Hospital Room of the Future

This section describes the design, deployment and initial set-up of our hospital room of the future to obtain a suitable dataset that allows us to perform some experiments. These experiments are aimed to measure the performance of the detection/classification methods as well as the time required to detect and mitigate different ransomware attacks. The designed scenario has the following elements:

- Five medical devices able to acquire different vital signs of patients and apply treatments. The medical devices are simulated using ICE Equipment Interfaces of the OpenICE v1.0.0 [17] software and each one of them runs on top of different machines with networking capabilities. The five machines have different operating systems for the sake of diversity. Specifically, we combine one MS Windows 7, vulnerable to different ransomware families explained below in detail, with four patched machines (two MS Windows 10, and two Ubuntu 16.04).
- One clinical database storing medical data such as EHR. The database is distributed across the machines with MS Windows. Additionally, there is a clinical information system in charge of reading and writing the database content through the SMBv1 protocol of Microsoft.
- One closed-loop application [43] implemented by the ICE Supervisor of the OpenICE software. The medical application runs on a dedicated machine with a vulnerable MS Windows 7 and it is able to acquire medical data from the medical devices and the database, analyse the data, and suggest medical treatments over medical devices.
- The Monitoring module of the ICE++ architecture. This module has been deployed on a dedicated machine with Ubuntu 16.04 that receives all the ICE network traffic. To implement the flow exporter, we have used Argus v3.0.8.1 [44], which is one of the most popular tools for network flow acquisition. On the other hand, the flow collector component has been designed and implemented by us using the Python language. The flow collector uses NumPy to compute efficiently the feature vectors from the stream of flows provided by the flow exporter.
- The Analyser module of the ICE++ architecture has been deployed in a dedicated machine with Ubuntu 16.04. The Anomaly Detection module uses Scikit-learn v0.20.0, which provides efficient implementations of the three evaluated ML methods. Similarly, the Ransomware Classification module, based on Naive Bayes, has been implemented also in Scikit-learn.
- The Decision and Reaction components of ICE++ runs on another Ubuntu 16.04 machine and we have used SWRL (Semantic Web Rule Language) [45] to define the management rules and SPARQL [46] queries to obtain the rules decision.
- One SDN-enabled router in charge of managing the communications of the previous elements through a private network. In addition, the router also enables some external communication through the Internet to allow doctors and caregivers to control remotely specific components of the clinical scenario.

The previous elements were fully virtualized in a physical server where OpenStack [47] and OpenDaylight [48] run. We used OpenStack as VIM (view Figure 5) to deploy and instantiate the virtual machines hosting the ICE Equipment Interfaces, ICE Supervisor, database and the components of the ICE++ architecture. On the other hand, we used OpenDaylight as SDN Controller to control the router of our network topology. We used a VNFM as mobile edge platform manager and Open Baton [49] as orchestrator to schedule the decision made by the ICE++ architecture and communicate with the previous elements.

Once our scenario was designed and deployed, the next step was to test our detection and mitigation solution in this scenario. To this end, we decided to select four of the most dangerous and recent malware affecting clinical environments. Specifically, three ransomware strains (WannaCry, Petya and BadRabbit) and one cryptomining malware (PowerGhost). Once selected, we used two of them (WannaCry and Petya) to train our solution, and the other two to test its generalization power. WannaCry [35] is a well-known ransomware and its behaviour was deeply described in Section 3.2. Petya [36] also spreads itself by exploiting the EternalBlue vulnerability, but it deploys a more sophisticated two-stage attack. When a device is infected, Petya attempts to infect the vulnerable devices in the same network and stores a micro-kernel in the MBR of the main disk. After that, it reboots the computer and starts encrypting the whole hard drive. Similarly to Petya, BadRabbit [50], uses a two-stage mechanism, but it leverages a similar vulnerability of SMBv1 called EternalRomance. Finally, PowerGhost [51] is a cryptocurrency miner that uses fileless techniques. Unlike ransomware, the goal of a cryptominer is to remain hidden in the target system mining cryptocurrencies. However, despite the fact that it is not a ransomware, we decided to select it because it also exploits EternalBlue vulnerability to spread itself across the network.

Once the ransomware selection was performed, the next step was to create suitable datasets to later measure the performance of our detection and classification solution. For that, we captured six hours of clean network traffic from our hospital room of the future equipped with ICE. Then, we cloned four times our scenario. The ICE Supervisor machine of each scenario was infected with a different ransomware (WannaCry, Petya, BadRabbit and PowerGhost). The traffic generated in each ICE scenario during the ransomware spreading phase was captured in a different dataset. In the end, we had five datasets, one with the clean traffic dataset and four with the ransomware traffic. These datasets are one of the relevant contributions of this paper and they can be downloaded in [15]. The amount of traffic captured was different in each dataset because it was determined by the behaviour of the ransomware spreading phase during the 30 min of traffic acquisition (enough time to infect all the vulnerable devices). After that, the five datasets were sent to our flow exporter to be converted into netflows. These netflows were properly labelled and sent to our flow collector. This component computed the five final feature vector datasets: one for the clean traffic, and one for each of the four ransomware samples. In our proposal, the feature vectors are computed from a slicing flow window and are labelled according to the last flow included in the window; therefore, the size of this window is one of the hyper-parameters of our system that had to be tuned. To include this variable, the vector generation had to be performed for six window sizes (5, 10, 20, 30, 60 and 120 s) per each ransomware, generating a total of 24 datasets.

Finally, it is well-known that some anomaly detection techniques do not work efficiently with large amounts of samples. Therefore, to avoid this restriction from affecting the technique selection process, we made up each dataset by taking all the anomalous samples (50,537) plus 100,000 randomly extracted clean samples (approximately twice the anomalous set size), keeping the original order. In this way, we also reduce the imbalance of the dataset significantly. Each dataset was then partitioned into three contiguous subsets: training (80%), validation (10%) and test (10%). Usually, each subset of this partitioning is created by a random selection from the original dataset; however, in our case, the high correlation between close vectors made the contiguous split more appropriate. The resulting datasets were subsequently cleaned, and an unsupervised feature selection, consisting of dropping highly correlated features, was carried out as explained in Section 4.2.1.

### 6.2. Anomaly Detection Technique Selection

This section performs an experiment to select the most appropriate anomaly detection method. Considering the five datasets acquired and explained in the previous section and the three potential anomaly detection methods listed in Section 4.2.2, our goal was to determine which method provided the best performance for our hospital room of the future.

The first step was to select a set of representative anomaly detection techniques: OC-SVM, LOF and IF. Each one of these algorithms detects anomalies by attempting a different strategy. OC-SVM is a boundary-based technique and its description can be found in Section 4.2.2. LOF, in turn, is a density-based scheme for anomaly detection. For each point, a Local Outlier Factor is computed as the average of the ratio of the local density around the point and the local density of its k-nearest neighbours. Finally, IF is based on the concept of isolation tree, which is a randomly generated binary tree where the instances are recursively partitioned. In these trees, an anomaly needs less partitions to become isolated. When a forest of random isolation trees collectively produce shorter paths for a particular point, it is likely to be anomalous.

Every ML training procedure requires a feature engineering stage. In semi-supervised anomaly detection methods, only unsupervised feature selection procedures can be used (e.g., PCA). Otherwise, the feature selection process would be biased by the set of known ransomware, and it might discard features useful in future scenarios. This is the main reason why we did not select a subset of features by using our labelled dataset.

In order to calculate which of the previous three algorithms has the best detection performance, a metric of such performance should be defined. Given that anomalies are supposed to be rare, the accuracy is not considered a representative performance metric. In anomaly contexts, precision, recall and F1-score give more information about the detection performance of a model. To define these metrics, some concepts should be previously defined:

- True Positive (TP): Number of anomalies correctly detected.
- True Negative (TN): Number of normal samples correctly classified as normal.
- False Positive (FP): Number of normal samples incorrectly detected as anomalies.
- False Negative (FN): Number of anomalies incorrectly classified as normal.

Below, we show the metrics proposed to measure the anomaly detection technique performance. F1-score is the harmonic average of precision and recall. Its result is closer to the lowest of the two values; therefore, it includes information about how balanced precision and recall are. In anomaly detection problems, both precision and recall are important. A low precision score tells us that we are generating a large number of false positives, and a low recall indicates that we are missing many anomalies:

- Precision, indicates what percent of the predicted anomalies were actual anomalies,
  $$\mathrm{Precision}=\frac{\mathrm{TP}}{\mathrm{TP}+\mathrm{FP}},$$
- Recall or sensitivity, defines what percent of actual anomalies where predicted,
  $$\mathrm{Recall}=\frac{\mathrm{TP}}{\mathrm{TP}+\mathrm{FN}},$$
- F1-score, shows the trade-off between the precision and recall regarding the anomaly class,
  $$\text{F1-score}=2\times \frac{\mathrm{Precision}\times \mathrm{Recall}}{\mathrm{Precision}+\mathrm{Recall}},$$
- False Positive Rate (FPR),
  $$\mathrm{FPR}=\frac{\mathrm{FP}}{\mathrm{FP}+\mathrm{TN}}.$$

A key aspect of the training of every ML algorithm is the hyper-parameters optimization. Specifically, in our clinical environment, there are two sets of hyper-parameters: one of them associated with the anomaly detection model, and the other related to the classification model. In both cases, a set of hyper-parameters was selected to be tuned. Then, a grid search in the hyper-parameter space was carried out in order to figure out which configuration achieved the best performance. With regard to our anomaly detection algorithm, a list of the considered hyper-parameters can be found in Table 4. Every configuration of them was used to train our three algorithms with the clean traffic dataset. The fitted model was then validated with both the ransomware and clean validation datasets. An interesting advantage of using features based on statistical information of the flows in a time window is that the connection of a new device with a network traffic pattern similar to the rest does not have an impact on the detection performance.

**Figure 7:**
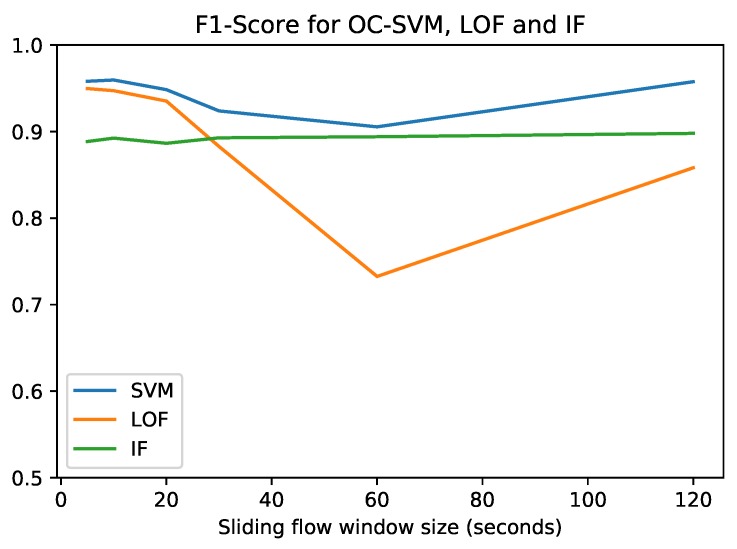
Performance of the best anomaly detection method configuration for each window size in the flow collector. The selected configuration for each method is the one that obtained the highest F1-score among all the configurations that properly detected at least 50% of each ransomware.

The performance results are summarized in Figure 7, where the best performance achieved by each anomaly detection method for each sliding flow window size is plotted. Figure 7 shows that OC-SVM obtains the maximum F1-score (0.9596), while the corresponding precision, recall and FPR values can be found in Table 5. This maximum is reached at a window size of 10 s. However, at this stage, the optimum flow sliding window size cannot be determined because it also depends on the ransomware classifier. Due to its satisfactory performance and stability with respect to the flow windows size, OC-SVM was the selected anomaly detection method to be used in our integrated clinical environment. Conversely, LOF was discarded due to its erratic behaviour. LOF has an extremely different performance depending on the flow window size. Due to the fact that our system shares the same sliding flow window between the anomaly detector and the ransomware classifier, a stable method is desirable. Finally, IF shows a really stable behaviour, but it was overcome by OC-SVM for each window size.

### 6.3. Ransomware Classifier Selection

Besides the anomaly detection method, our system also considers a ransomware classifier. This section focuses on showing the experiments focused on selecting the most appropriate classifier in terms of performance.

Three probabilistic well-known supervised classifiers were selected to be evaluated: Neural Network, Naive Bayes and Random Forest. Following the same methodology and metrics followed in the anomaly detector selection, the three algorithms were trained and validated. The selection process with these models was performed using only three datasets: Clean, WannaCry and Petya. The corresponding training and validation subsets were used to train the models in the hyper-parameter tuning phase. A grid search in the hyper-parameters allowed us to find the configuration providing the best performance for each model. The list of hyper-parameters considered is showed in Table 6.

It should be noticed that there is a hyper-parameter called Feature Selection. Since our classification dataset is properly labelled, we can do a more advanced feature selection by means of a Random Forest (RF). Before the training procedure, an RF is trained to obtain an estimation of the importance of each feature in the classification process. This importance score allows us to drop every feature with an importance below a given threshold (as was also described in Section 4.2.1). In our case, this threshold is considered one hyper-parameter and it is tuned with the rest. By increasing this threshold, the number of selected features is reduced, influencing in the performance of the ML algorithms. Then, the three models were evaluated with the validation subset of all the datasets (Clean, WannaCry and Petya, used in the training phase, and BadRabbit and PowerGhost, unknown for the classifier). The winner in the validation was the model with highest F1-score that, at least, detected properly 50% of every category. Once the model was chosen, the real performance was obtained with all the test subsets separately.

Table 7 shows the performance of the aforementioned chosen models for each configuration of hyper-parameters and ransomware with the test datasets. As a special case, the hyper-parameters of the neural network related to the number and size of layers have been omitted due to limitation of space. Given both a flow sliding window size and a threshold used in the feature selection procedure, their corresponding row in the Table 7 lists the classification accuracy of the five test datasets (Clear Traffic, WannaCry, Petya, BadRabbit and PowerGhost) for each of the three trained models. Despite the unbalanced datasets, accuracy is in this case an appropriate metric because it was computed separately for each class.

As a conclusion of this experiment, we can state that, although the three evaluated algorithms achieve excellent accuracy, Naive Bayes is the algorithm that reaches the best performance, precisely with the flow window size of 10 s (highlighted in grey background in Table 7). This window size coincides with the best-performing window size in the anomaly detection experiment. Interestingly, the feature selection threshold has little or no impact in the results of the winner algorithm. Additionally, Table 7 demonstrates the importance of this stage of grid search of hyper-parameters in order to find a model that accurately classifies the two unseen ransomware samples. As an illustration, the same winner Naive Bayes configuration trained with a flow window size of 120 s instead of 10 s can hardly classify BadRabbit samples. Similarly, just changing the classification method with the same window size and feature selection threshold can result in worse performance. For example, Random Forest with the same window of 10 s and the same thresholds obtained significantly less performance than Naive Bayes in Petya and BadRabbit classification.

Another interesting aspect of this experiment is that it has shown that BadRabbit has a pattern traffic behaviour similar to Petya, since it was always identified as Petya ransomware. Similarly, PowerGhost was always classified as WannaCry, indicating that their propagations are similar at some extent.

### 6.4. Anomaly Detection and Classification Time

This section shows the experiments performed with the goal of measuring the time needed by our solution to detect and classify ransomware affecting our hospital room of the future equipped with ICE. Once both the anomaly detector (OC-SVM) and the classifier (Naive Bayes) are selected, another critical aspect is to measure how much time these techniques need in order to evaluate, in real time, a current feature vector and decide whether it is an anomaly or not. At this point, it is important to remember that both processes (anomaly detection and classification) are performed in parallel and their outputs are subsequently combined by the rules defined in Section 4.4.1.

Regarding the anomaly detection system deployed in the analyser module of our ICE++ architecture (Section 4.3), we measured the time consumed by the OC-SVM in performing 100 evaluations of different feature vectors. We obtained that OC-SVM required 0.006 s on average to evaluate a unique feature vector. In a similar way, we performed another experiment involving 100 evaluations using the Naive Bayes algorithm, which was deployed also in the analyser component of ICE++. The experiment returned that Naive Bayes required 0.022 s on average to classify an individual feature vector. Since both processes are performed in parallel, we consider the longest lapse as the time required to detect and classify a ransomware in our scenario. Finally, we had to add the 10-s delay imposed by the timeout of the flow exporter, mainly due to the UDP flows. The main conclusion of the experiment is that the detection and mitigation time (10.0221 s) is not only acceptable but also insignificant when compared with the time required by the ransomware to spread around the ICE network (see Section 6.6).

### 6.5. Mitigation Reaction Time

This section demonstrates the suitability of the proposed solution when a ransomware, affecting our hospital room of the future equipped with ICE, needs to be mitigated. Specifically, we have performed several experiments with the goal of measuring the time required by the ICE++ architecture to deploy, launch and configure an ME application with a new ICE Equipment Interface of OpenICE that simulates a medical device (steps 2 and 5 of Figure 6).

To deploy and configure the ICE Equipment Interface, we used the following three different configurations of hardware:

- Raspberry Pi 3 Model B with 1 GB of RAM and a Quad Core ARM Cortex-A53 CPU at 1.2 GHz hosting openSUSE Leap 42.3 as an operating system;
- Personal computer with 16 GB of RAM and Quad Core Intel(R) CPU i7-3770 at 3.40 GHz. The operating system is Ubuntu 16.04 LTS Desktop; and
- Server with 64 GB or RAM, a 36 Core Intel(R) Xeon(R) CPU E5-2697 v4 at 2.30 GHz, and Ubuntu 16.04 LTS Server as an operating system.

For each one of the previous hardware configurations, we developed and launched an OpenICE instance using VM and containers. The first experiment focused on measuring the time required to deploy the ICE Equipment Interface on top of different VMs. For that purpose, we followed the next steps:

1. Creation of three VM with 1 GB of RAM, a single-core CPU, and 10 GB of hard disk, each one of them using OpenStack.
2. Installation of three operating systems with different capabilities (openSUSE Leap 42.3, Ubuntu 16.04 LTS Server, and MS Windows 7), each in its own VM.
3. Installation and configuration of the ICE Equipment Interface simulating a medical device, on top of the previous operating systems.
4. Measurement of the time required to instantiate each previous VMs in each piece of hardware equipment.
5. Measurement of the time required to launch and configure a simulated medical device in the ICE Equipment Interface running on top of the hardware equipment.

At this point, it is important to clarify that, due to the limited computational resources of the Raspberry Pi 3, this device was not able to host virtual machines.

After performing the previous experiment with VMs, we carried out an additional experiment with containers. Specifically, we deployed the ICE Equipment Interface of OpenICE in a Docker that uses *Java SE Runtime Environment 8* (library required by OpenICE) to execute the version 0.7.0 of OpenICE. After implementing and configuring the Docker, we measured the time required to deploy and launch a simulated medical device of OpenICE. Table 8 shows the times obtained for the deployment and initiation of the ICE Equipment Interface running on top of different virtualization techniques and hardware configurations.

One of the main conclusions obtained after performing the previous experiments is that the time required to deploy the ICE Equipment Interface is inversely proportional to the amount of computational resources needed. However, it is important to keep a trade-off between resources and economical cost. Therefore, the Raspberry Pi 3 is not suitable for real-time medical scenarios where ransomware spreads quickly, and medical devices must be deployed considering critical time restriction. In contrast, medical scenarios with other requirements in terms of latency can be perfectly managed by the Raspberry Pi 3. Another conclusion we can extract from Table 8 is that the deployment time is much lower using Dockers than with virtual machines. It makes sense because containers are computationally lighter than VMs, since they do not need to boot their own operating system. However, it is important to consider that, if the software to control the medical devices and services is implemented in MS Windows, virtual machines are a better alternative because containers are oriented to Linux operating systems. Furthermore, containers are also more exposed to attack vectors than virtual machines are. Therefore, using containers requires to take actions to secure them, such as reducing users’ privileges or running services as a non-root user.

### 6.6. Infection and Mitigation Time

This section demonstrates that our solution is able to detect and mitigate the previous families of malware (WannaCry, Petya, BadRabbit and PowerGost) in an acceptable time. We consider as acceptable any time lower than the required by the ransomware to spread across ICE and infect the second medical device or machine.

Table 9 shows the best and worst detection and mitigation cases (in terms of time) for the four selected ransomware samples. It is important to note that the time differences between these two cases are influenced by the hardware and virtualization techniques used during the mitigation phase (more details in Table 8). Specifically, we measured 15.885 s during the deployment and configuration of a Docker in a Raspberry Pi 3. In contrast, when we deployed and configured the new ICE element in the Server using Dockers, the measured time was 2.434 s. It should be also noted that the detection and classification time is influenced by the 10-s timeout of the Flow Exporter.

**Figure 8:**
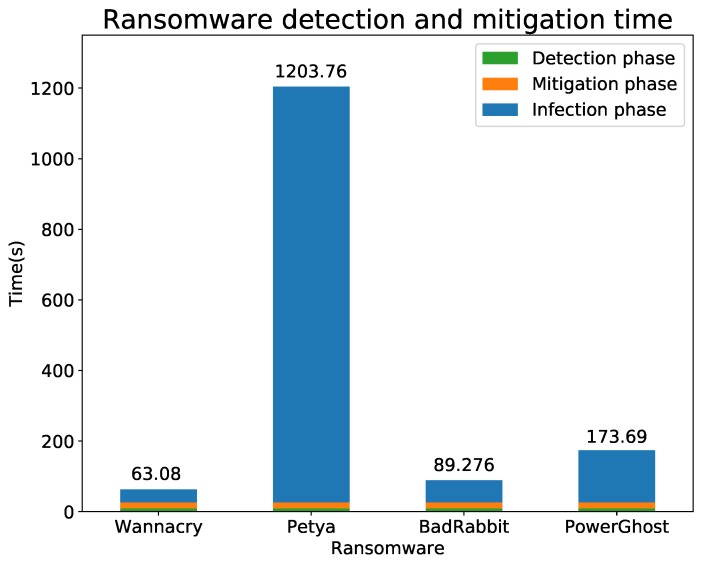
Comparison between the times required by the detection, mitigation and infection phases of ransomware. Detection and mitigation phases are running sequentially, whereas the infection phase goes in parallel to the previous two.

Figure 8 plots a comparison between the calculated detection and mitigation times shown in Table 9 and the times required by WannaCry, Petya, BadRabbit and PowerGost to infect our ICE. To measure the infection time for each ransomware, we analysed the ICE network traffic (acquired by the Monitoring component of our ICE++ Architecture), and identified the first network package sent by the first and second infected devices. After that, we obtained their time stamps and calculated the difference between them.

As a conclusion, we have demonstrated that our intelligent and automatic solution is suitable for detecting and mitigating the spreading phase of different ransomware families in real time. In this context, the performed experiments provide promising results in terms of time and precision to avoid different ransomware from spreading across the integrated clinical environments.

## 7. Conclusions and Future Work

This paper presents an automatic, intelligent and real-time system able to detect, classify, and mitigate ransomware attacks in hospital rooms of the future. The proposed solution is fully integrated with the ICE++ architecture, our previous work, and makes use of Machine Learning techniques to detect and classify the spreading phase of ransomware attacks affecting ICE. Another relevant contribution of this paper is the proposed mitigation mechanism, which considers NFV/SDN paradigms to stop the spreading by isolating and replacing infected medical devices and systems.

On the other hand, a pool of experiments have demonstrated the suitability of our solution detecting some of the most recent and dangerous malware (WannaCry, Petya, BadRabbit and PowerGhost). The selected techniques, OC-SVM and Naive Bayes, have proved able to detect and classify ransomware affecting ICE. OC-SVM obtained the 92.32% of precision and 99.97% of recall in anomaly detection, whereas Naive Bayes obtained a 99.99% of classification accuracy. Both of them were evaluated with known and unseen ransomware samples. Additional experiments demonstrated the viability of the proposed solution in terms of time. In the worst case, our solution detected and mitigated a ransomware attack in 29.7 s, which is acceptable because the ransomware required at least 63.1 s to spread and infect other devices. Finally, different labelled ransomware datasets in ICE have been created and made publicly available.

As the next step, we are already working on the evaluation of our proposal with new ransomware families having more complex spreading behaviour patterns. In this context, the inclusion of new anomaly detection and classification techniques will be required to maintain the accuracy level obtained as output of this work. Additionally, we plan to extend our solution and analyse the encryption of shared folders, where the ransomware traffic patterns could be seen as time series data and classified by deep learning methods like Long Short-Term Memory neuronal networks (LSTM). We will also include ML techniques to detect the infection/encryption ransomware stages by means of particular system call patterns and internal processes executed on the medical devices. The combination of the solution proposed in this paper (focused on detecting the ransomware spreading phase) together with the analysis of the infection/encryption phase will improve the robustness and accuracy of our detection system. Another line of work that we are considering is the automatic generation of signatures and metadata to feed existing countermeasures such as IDS and anti-virus in real time and avoiding massive ransomware from spreading.

## Figures and Tables

**Table 1:** Comparison of our work with other relevant proposals.

Reference	Target	Context	Input	Technique	Traffic	Mitigation
[25]	Ransomware	C&C Connections	IP Header	Blacklist	Cypher	SDN
[26]	Ransomware	C&C Connections	HTTP length	Classifier	Plain	SDN
[27]	Ransomware	C&C Connections	Netflows	Classifier	Cypher	–
[28]	Malware	Mobile	HTTP & Netflows	Classifier	Plain	–
[30]	Malware	Network	Netflows	Classifier	Cypher	–
[31]	Ransomware	App execution	Syscalls	Classifier	Cypher	–
[32]	Ransomware	Network	Netflows	Classifier	Cypher	–
Our solution	Ransomware	Integrated Clinic Environment	Netflows	Anomaly & Classifier	Cypher	SDN/NFV

C&C: Command and Control; SDN: Software Defined Networking; NFV: Network Function Virtualization.

**Table 2:** Flow record fields obtained from the traffic captured.

Start time Flow duration (s) Protocol (UDP/TCP/ARP) Source IP Destination IP Source port Destination port Direction State	Total packets Source packets Total bytes Source bytes Total load (bits/s) Source load (bits/s) Source inter-packet arrival time (msec) Destination inter-packet arrival time (msec)

**Table 3:** TCP/UDP features computed from the network flows.

Total Features	ARP	Last Flow ^1^	TCP/UDP × All Directions ^2^	TCP/UDP × Internal/External Incoming/Outgoing ^3^	Features
11	1		2	8	Number of flows.
8				8	% of flows.
18	2			16	Mean and stddev of the flow durations.
18	2			16	Mean and stddev of time between two consecutive flows.
8				8	Number of different destination IPs.
8				8	Entropy of destination IPs.
60			12	48	Sum, max, min, mean, stddev and median of total packets.
60			12	48	Sum, max, min, mean, stddev and median of source packets.
60			12	48	Sum, max, min, mean, stddev and median of total bytes.
60			12	48	Sum, max, min, mean, stddev and median of source bytes.
60			12	48	Sum, max, min, mean, stddev and median of total load.
60			12	48	Sum, max, min, mean, stddev and median of source load.
16				16	% of source/destination ports >1024.
16				16	% of source/destination ports <1025.
16				16	Number of different source and destination ports.
16				16	Entropy of source and destination ports.
1	1				Number of different destination IPs.
1	1				Entropy of destination IP.
1	1				Median of the duration.
3		3			Protocol used (TCP, UDP, ARP).
4		4			State (INT, RST, FIN, CON).
2		2			Source or destination port <1024.
1		1			Destination port.
4		4			Direction(incoming, outgoing, internal, external).
8		8			Total packets, source packets, total bytes, source bytes, load, source load, source mean interpacket arrival time and destination mean interpacket arrival time.
**520**					**Total features computed.**

${}^{1}$ Extracted from the last flow; ${}^{2}$ Computed per protocol; ${}^{3}$ Computed per pair (protocol, direction).

**Table 4:** Hyper-parameters tuned in the selection process of the the anomaly detection technique.

Hyper-Parameter		List/Range of Values
Flow Collector	window (s)	[5, 10, 20, 30, 60, 120]
OC-SVM	nu	[0.0001, 0.0005, 0.001, 0.005, 0.01]
	gamma	[0.0001, 0.0005, 0.001, 0.005, 0.01]
LOF	neighbours	[10, 20, 30, 50]
	leaf size	[10, 20, 30, 40]
	contamination	[0.1, 0.05, 0.01, 0.005]
IF	estimators	[100, 200, 300]

OC-SVM: One-Class Support Vector Machine; LOF: Local Outlier Factor; IF: Isolation Forest.

**Table 5:** F1-Score, precision, recall and false positive ratio (FPR) values of OC-SVM for a sliding flow window size of 10 s.

F1-Score	Precision	Recall	FPR
0.9596	0.9232	0.9997	0.046

**Table 6:** Hyper-parameters tuned in the ransomware classifier selection process.

Hyper-Parameter		List/Range of Values
Flow Collector	window (s)	[5, 10, 20, 30, 60, 120]
Feature Selection	importance	[0.001, 0.0005, 0.0002, 0.0001]
Neural Network	First layer	[8, 10, 16]
	Second layer	[0, 4, 6]
Gaussian Naive Bayes	no parameters	
Random Forest	estimators	[100, 200, 300]

**Table 7:** Classification accuracy of each model for different configurations of hyper-parameters and ransomware. C: Clean, W: WannaCry, P: Petya, B:BadRabbit, G: PowerGhost.

Window Size	Feat. Sel. Threshold	Neural Network					Naive Bayes					Random Forest				
		C	W	P	B	G	C	W	P	B	G	C	W	P	B	G
5 s	0.0001	0.9997	1.0000	1.0000	0.9773	0.9991	0.9870	1.0000	1.0000	0.9975	0.9999	1.0000	1.0000	1.0000	0.9798	0.9998
	0.0002	0.9999	1.0000	1.0000	0.9748	0.9989	0.9871	1.0000	1.0000	0.9975	0.9999	1.0000	1.0000	1.0000	0.9798	0.9985
	0.0005	0.9998	1.0000	1.0000	0.9572	0.9990	0.9900	1.0000	1.0000	0.9975	0.9999	1.0000	1.0000	1.0000	0.9899	0.9998
	0.0010	1.0000	1.0000	0.9596	0.9723	0.9985	0.9839	1.0000	1.0000	1.0000	1.0000	1.0000	1.0000	1.0000	0.9924	1.0000
10 s	0.0001	0.9993	1.0000	1.0000	0.9923	1.0000	**0.9900**	**1.0000**	**1.0000**	**1.0000**	**0.9999**	0.9999	1.0000	0.9773	0.9846	0.9998
	0.0002	0.9994	1.0000	1.0000	0.9949	0.9997	**0.9903**	**1.0000**	**1.0000**	**1.0000**	**0.9999**	0.9999	1.0000	0.9773	0.9871	0.9998
	0.0005	0.9988	1.0000	1.0000	0.9949	0.9990	**0.9897**	**1.0000**	**1.0000**	**1.0000**	**1.0000**	0.9999	1.0000	0.9886	0.9949	0.9998
	0.0010	0.9995	1.0000	1.0000	0.9974	0.9979	0.9840	1.0000	1.0000	1.0000	1.0000	0.9999	1.0000	0.9773	0.9923	1.0000
20 s	0.0001	1.0000	1.0000	0,9888	0.9811	1.0000	0.9970	1.0000	0.9875	0.9622	0.9999	1.0000	1.0000	0.9875	0.9703	0.9999
	0.0002	1.0000	1.0000	1.0000	0.9703	1.0000	0.9985	1.0000	0.9750	0.9568	0.9999	1.0000	1.0000	1.0000	0.9730	0.9999
	0.0005	1.0000	1.0000	1.0000	0.9919	1.0000	0.9961	1.0000	0.9875	0.9622	0.9999	1.0000	1.0000	0.9875	0.9730	0.9999
	0.0010	1.0000	1.0000	1.0000	0.9784	0.9999	0.9933	1.0000	0.9875	1.0000	1.0000	1.0000	1.0000	0.9875	0.9784	0.9999
30 s	0.0001	0.9999	1.0000	1.0000	0.9945	0.9998	0.9986	1.0000	1.0000	0.9727	0.9999	1.0000	1.0000	1.0000	0.9863	0.9999
	0.0002	1.0000	1.0000	0,9885	0.9809	0.9999	0,9988	1.0000	1.0000	0.9781	1.0000	1.0000	1.0000	1.0000	0.9809	0.9999
	0.0005	1.0000	1.0000	0.9885	0.9809	0.9899	0.9992	1.0000	1.0000	0.9891	1.0000	1.0000	1.0000	1.0000	0.9836	0.9999
	0.0010	1.0000	1.0000	0.9885	0.9836	0.9999	0.9961	1.0000	0.9873	0.9727	1.0000	1.0000	1.0000	1.0000	0.9781	0.9999
60 s	0.0001	1.0000	1.0000	0.9880	0.6296	0.9999	0.9986	1.0000	0.8784	0.6866	0.9999	1.0000	1.0000	0.9865	0.9601	0.9999
	0.0002	1.0000	0.9996	1.0000	0.4986	0.9999	0.9993	1.0000	1.0000	0.0000	0.9999	1.0000	1.0000	0.9865	0.9573	0.9999
	0.0005	1.0000	1.0000	1.0000	0.7949	0.9999	0.9996	1.0000	0.9324	0.3333	0.9999	1.0000	1.0000	1.0000	0.9601	0.9999
	0.0010	1.0000	1.0000	0.9880	0.6296	0.9999	0.9986	1.0000	0.8784	0.6866	0.9999	1.0000	1.0000	0.9865	0.9601	0.9999
120 s	0.0001	0.9999	1.0000	1.0000	0.6034	0.9999	0.9996	1.0000	0.9855	0.0170	0.9999	1.0000	1.0000	1.0000	0.2975	0.9999
	0.0002	1.0000	1.0000	1.0000	0.7054	0.9999	0.9997	1.0000	0.9855	0.0170	1.0000	1.0000	1.0000	1.0000	0.3569	0.9999
	0.0005	1.0000	1.0000	1.0000	0.8895	0.9999	0.9999	1.0000	0.9855	0.0255	1.0000	1.0000	1.0000	1.0000	0.3173	0.9999
	0.0010	1.0000	1.0000	1.0000	0.9490	0.9999	0.9989	1.0000	0.9855	0.3201	0.9999	1.0000	1.0000	1.0000	0.3484	0.9999

**Table 8:** Deployment time of a new ICE Equipment Interface with a virtual medical device.

Hardware	Container Deployment	Virtual Machine Deployment			OpenICE Instantiation and Configuration
**Details**	**Java**	**openSUSE Leap 42.3**	**Ubuntu 16.04**	**MS Windows 7**	**ICE Equipment Interface**
Raspberry Pi 3	7.348 s	-	-	-	8.537 s
Personal Computer	0.460 s	6.731 s	7.164 s	13.446 s	2.802 s
Server	0.314 s	4.774 s	5.902 s	10.523 s	2.120 s

**Table 9:** Worst and best cases of ransomware detection and mitigation times.

	Detection and Classification	Mitigation	Total
Worst case	10.022 s	15.885 s	23.468 s
Best case	10.022 s	2.434 s	10.456 s
